# Synergistic Interaction Between HPV‐16 E7 Oncoprotein and Severe Vitamin A Deficiency in Regulating Adaptive Immunity in a Preclinical Cervical Cancer Model

**DOI:** 10.1002/jmv.70992

**Published:** 2026-06-10

**Authors:** Armando Chávez‐Ríos, Celina López‐Ruiz, Ian A. García‐Aguirre, Rodolfo Ocadiz‐Delgado, Francisco Garcia‐Sierra, Rogelio Hernández‐Pando, Solangy Lizcano‐Meneses, Patricio Gariglio

**Affiliations:** ^1^ Department of Genetics and Molecular Biology Centro de Investigación y de Estudios Avanzados del Instituto Politécnico Nacional (Cinvestav‐IPN) Mexico City Mexico; ^2^ Departamento de Ingeniería en Biotecnología, División de Ciencias de Salud, Biológicas y Ambientales Universidad Abierta y a Distancia de México Mexico City Mexico; ^3^ Departamento de Bioingeniería, Escuela de Ingeniería y Ciencias Tecnologico de Monterrey Ciudad de México Mexico; ^4^ Department of Cell Biology Centro de Investigación y de Estudios Avanzados del Instituto Politécnico Nacional (Cinvestav‐IPN) Mexico City Mexico; ^5^ Department of Pathology Instituto Nacional de Ciencias Médicas y Nutrición “Salvador Zubirán” Mexico City Mexico

**Keywords:** cervical cancer, HPV‐16 E7 oncoprotein, immune dysregulation: Vitamin A deficiency, Vitamin A‐normal diet reintegration

## Abstract

Persistent infection with High‐Risk Human Papillomavirus (HR‐HPV), particularly HPV‐16, is the main driver of cervical intraepithelial neoplasia (CIN) and cervical cancer (CC). However, HR‐HPV infection alone is insufficient for malignant progression and nutritional cofactors such as Vitamin A deficiency, may influence cervical neoplasia progression through immune dysregulation. This study aimed to explore the interaction between HPV‐16 E7 oncoprotein expression and severe Vitamin A Deficiency (VAD), as well as the effects of a Vitamin A‐Normal Diet Reintegration (NDR) on adaptive immune regulation in cervical neoplasia using the K14E7 preclinical murine model. We found that VAD worsening cervical epithelial changes and impairs the cytotoxic ability of CD8^+^ T cells, which is marked by lower levels of granzyme B and increased expression of PI‐9. These changes were accompanied by increase F4/80^+^ macrophage infiltration and elevated Foxp3 expression, suggesting enhanced Tregs immunosuppressor activity. Distinctly, NDR induced partial recovery of cervical epithelium architecture and the effector function of CD8^+^ T cells. Our results highlight Vitamin A as a key immunomodulator that influences the balance between immune surveillance and immune evasion of HPV‐associated cervical carcinogenesis and suggests its potential relevance for preventive or adjuvant treatments in populations at high risk of CC.

## 1 Introduction

Persistent human papillomavirus (HPV) infection, especially high‐risk types like HPV‐16, is a major risk factor for the development and progression of CC, which remains one of the leading causes of mortality in women worldwide. One of the key mechanisms in the progression of cervical epithelium toward malignancy is the overexpression of early HR‐HPV oncoproteins, specifically the E7 oncoprotein, which interferes with numerous transcriptional regulators and with the expression of proteins involved in cell cycle control, including the cyclin‐dependent kinase inhibitors p21 and p27, as well as cyclins A and E [1]. Furthermore, E7 promotes the degradation of retinoblastoma protein (pRb), a crucial tumor suppressor that regulates the transition from G1 to S phase of the cell cycle [2, 3]. Additionally, chronic inflammation is another hallmark influenced by E7 protein, which interacts with pattern recognition receptors such as Toll‐like receptor 9 (TLR9), leading to disruption of IFN‐γ signaling and inhibition of cyclic GMP‐AMP synthase, a key enzyme in detecting foreign DNA in the cytosol [4, 5].

**Figure 1:**
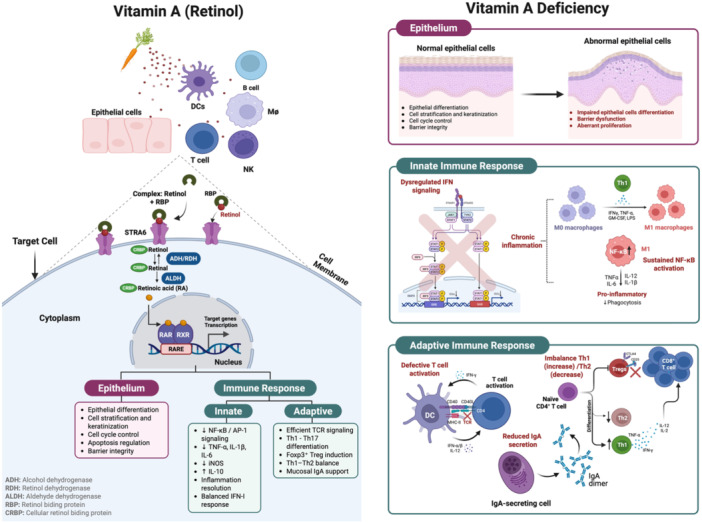
Schematic representation of Vitamin A (retinol)‐dependent regulation of epithelial differentiation and immune responses (innate and adaptive) and the biological consequences of Vitamin A deficiency.

Recent research has focused on uncovering other factors that may affect the progression of premalignant cervical lesions, with deficiencies in micronutrients like Vitamin A becoming increasingly important. Vitamin A (retinol) is an essential micronutrient needed in small amounts by the human body. In its retinoid form such as retinol, retinal, retinyl ester, or retinoic acid. Vitamin A is found in animal products or, alternatively, in fruits and vegetables as carotenoids, which are converted into retinol in the intestine and other tissues [6]. Retinoic acid (RA), the biologically active form of Vitamin A, regulates gene expression by binding to two types of nuclear receptors: retinoic acid receptors (RARs) and retinoid X receptors (RXRs). These receptors form heterodimers (RARs‐RXRs) that attach to retinoic acid response elements (RAREs) in DNA, thereby influencing the transcription of genes essential for cell proliferation, epithelial differentiation, and apoptosis [7, 8], as well as modulate immune response genes of both innate and adaptive immunity. Its effects on the innate immune response show that RA suppresses the NF‐κB and AP‐1 dependent transcription of pro‐inflammatory mediators, such as TNF‐α, IL‐1β, IL‐6, and inducible nitric oxide synthase (iNOS) [9]. At the same time, it promotes the production of IL‐10, aiding in the resolution of inflammation and regulating type I interferon to enhance early antiviral responses. RA is also implicated in shaping the activation and differentiation of CD4^+^ T helper cells and establishing a balance between the Th1 and Th2 responses [7]. It supports the differentiation of Th1 and Th17 cells during inflammatory responses and promotes the development of Foxp3^+^ regulatory T cells through enhanced TGF‐β‐Smad3 signaling [10, 11]. Scientific literature links vitamin A deficiency to cervical carcinogenesis in humans and mouse models, showing collectively, disruption of the vitamin A‐RA axis could contributes to impaired viral HPV control, chronic inflammation and viral persistence that result in histological changes such as hyperplasia, metaplasia, and cellular dysplasia that increase the risk of developing CC [8, 12]. (Figure 1)

Vitamin A and its derivatives, especially all‐trans‐retinoic acid (ATRA), play a wide range of modulatory roles in the innate and adaptive immune systems. Vitamin A deficiency leads to impaired proliferation and differentiation of immunosuppressive leukocytes due to the depletion of RA, which can increase the risk of developing cervical carcinoma [13]. ATRA functions as a regulator of formation of the epithelium, keratinization, stratification, cellular differentiation, recruitment, and polarization of immune cells within the tumor microenvironment, such as T cells, dendritic cells, and macrophages [14, 15].

Therefore, the main goal of our study was to assess the synergistic effect of HPV‐16 E7 oncoprotein expression with VAD and NDR on the modulation of the adaptive immune response in cervical neoplasia, using the K14E7 preclinical model. The K14E7 transgenic mouse, which expresses HPV16 E7 in basal keratinocytes, exhibits epithelial alterations such as hyperplasia and dysplasia that resemble those found in human cervical lesions [16], supporting its clinical relevance as a preclinical model of HPV‐associated carcinogenesis.

This approach provides insights into the immune mechanisms that drive the development and progression of CC under conditions of immunological and nutritional disruption.

## 2 Materials and Methods

### 2.1 Transgenic K14E7 and Non‐Transgenic FvB Female Mice

Transgenic K14E7 [16] and non‐transgenic FvB mice were provided by the animal facility of the Laboratory Animal Care (UPEAL), and Research Unit of the Center for Research and Advanced Studies of the IPN (Cinvestav), in compliance with the regulations of the Association for the Assessment and Accreditation of International Laboratory Animal Care (AAALAC). FvB females were obtained from non‐transgenic breeding, while K14E7 females were obtained from crossing K14E7 males with FvB females. All experiments and procedures were approved by the UPEAL‐CINVESTAV‐IPN Committee, in compliance with NOM‐062‐Z001999 (Protocol No. 0193‐16).

### 2.2 Generation of the Crosses

A 2‐ to 3‐month‐old FvB female mouse was bred into a 4‐ to 5‐month‐old K14E7 male mouse, both of which were on a Normal Diet (ND). From gestation onward, pregnant females were fed a Vitamin A‐deficient Diet (VAD) (Mod TestDiet 57W5 w/No Added Vitamin A, PMI Nutrition International, USA). A second breeding was performed under VAD conditions, and offspring from the second litter were used for the study. Control mice were maintained on ND, following the same crossing protocol and maintaining a standard diet from gestation until the second litter was obtained.

### 2.3 Dietary Regimens

Females FvB mice (control) and K14E7 transgenic mice were divided into three experimental groups (*n* = 3 per group), based on their diet from the first gestation to 3 months of age:

*Normal Diet (ND) group:* received a standard diet throughout gestation and until 3 months of age.
*Vitamin A‐Deficient Diet (VAD) group:* received a diet lacking vitamin A continuously from the first gestation until sacrifice at 3 months of age (second generation).
*Normal Diet Reintegration (NDR) group:* received a vitamin A‐deficient diet as in the VAD group but switched back to a standard diet 1 month before sacrifice.

The mice were anesthetized with isoflurane and euthanized by cervical dislocation at 3 months old. Cervical tissue was then extracted and fixed overnight in 4% paraformaldehyde.

### 2.4 Histopathology and Evaluation of the Degree of Epithelial Injury

The cervical tissues were embedded in paraffin, and serial 5‐µm sections were obtained using a HistoCore BioCut microtome (Leica Biosystems; Deer Park, USA). The tissue was then deparaffinized at 56°C and rehydrated through a graded series of organic solvents with increasing polarity. Specifically, slides were washed in 100% xylene and 100% EtOH for 15 and 5 min, respectively, followed by rinsing in distilled water and 1× PBS. Finally, the tissue sections were stained with hematoxylin and eosin using the kit Leica ST Infinity H&E Staining System (Leica Biosystems; Deer Park, USA) and the degree of epithelial injury was further evaluated by an expert pathologist (R.H.P.) (see Supplementary Table 1). The microphotography acquired at 20× was used for cervical area quantification.

### 2.5 Immunohistochemistry (IHC)

Cervical tissue sections mounted on slides were deparaffinized and rehydrated. IHC detection of Proliferating Cell Nuclear Antigen (PCNA), GzmB, PI‐9, CD4, and CD8 (see Supplementary Table 2), was performed. Antigen retrieval was conducted in 1× Citrate Buffer (BSB 0020, Bio SB, USA) using a pressure cooker. IHC was carried out using the BioGenex Super Sensitive Polymer‐HRP IHC Detection System (BioGenex; Arhnem, The Netherlands) according to the manufacturer's instructions. Slides were incubated overnight at 4°C with primary antibodies, using dilutions specified for each marker and secondary antibody conjugated to poly‐HRP was added. Signal was developed with DAB chromogen. Negative controls omitted the primary antibody. Tissues were counterstained with hematoxylin and covered coverslips were with Entellan mounting medium (Merck, Darmstadt, DE). Five random fields per tissue were analyzed at 20x and 40x magnification using a brightfield microscope (Nikon ECLIPSE 80i).

### 2.6 Immunofluorescence (IF)

For immunofluorescence, 5 μm‐thick paraffin‐embedded cervical tissue sections were deparaffinized at 56°C for 20 min and rehydrated. Antigen retrieval was performed as described for IHC. SSections were blocked and permeabilized (10 mg/mL BSA, 0.1% Triton X‐100, and 0.2% SDS) and incubated overnight at 4°C with the primary antibodies against FoxP3 and F4/80, according to the dilution specified for the marker (see Supplementary Table 3). For F4/80 marker, tissue sections were incubated with the secondary antibody (α‐rat 1:200, Invitrogen Thermo Fisher Scientific, Waltham, Massachusetts, USA) conjugated to the fluorophore fluorescein isothiocyanate (FITC) for 1 h. Then, followed by counterstaining with the fluorescent nuclear DNA dye Hoechst (1:10,000 dilution in 1× PBS + 0.1% Triton X‐100). Tissues were mounted in Vectashield and visualized using confocal microscope (Leica DMi8). Negative controls omitted the primary antibody.

### 2.7 Digital Analysis of IHC and IF Images

Digital micrographs were analyzed using QuPath version 0.5.1 software (University of Edinburgh, UK) to quantify cells positive for different IHC and IF markers in cervical tissues from the six experimental groups: FVB ND, FVB VAD, FVB NDR, K14E7 ND, K14E7 VAD, and K14E7 NDR. Five random fields per tissue were selected, and images were acquired at 20×, 40×, and 60× magnification and analyzed by manually selecting regions of interest (ROIs) encompassing the cervical epithelium and stroma. The software automatically detected cells and classified them as positive or negative based on signal intensity. For CD4^+^ and CD8^+^ T cells, membranous or cytoplasm brown staining was considered positive. For immunofluorescence images, thresholds were set based on fluorescence intensity (FITC/APC fluorochrome).

### 2.8 Statistical Analysis

Statistical analysis was performed using GraphPad Prism version 8.0.1 software (GraphPad Prism Inc. USA). Data were expressed as a percentage of positive cells (PCNA, CD4, CD8, GzmB, PI‐9, FoxP3, F4/80) and presented as mean ± SD. Data represent three independent experiments with three mice per group (*n* = 3). Comparisons were performed using two‐way ANOVA with genotype (FvB or K14E7) and dietary condition (ND, VAD, or NDR) as factors, followed by Tukey's post‐hoc test. Statistical significance was set at *p* < 0.05.

CD4^+^/CD8^+^ and GzmB^+^/PI‐9^+^ ratios were calculated for each mouse by dividing the percentage of positive cells.

## 3 Results

### 3.1 Normal Diet Reintegration (NDR) Restores Cervical Epithelial Architecture and Reduces Vad‐Induced Cervical Areas in FvB and K14E7 Transgenic Model

**Figure 2:**
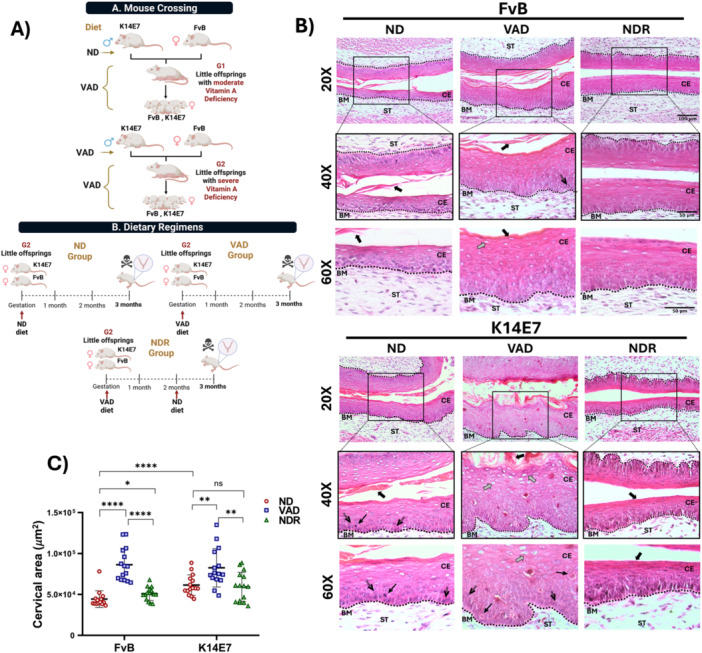
Histopathological analysis of ectocervical tissue sections obtained from 3‐month‐old non‐transgenic (FvB VAD, FvB NDR) and transgenic mice (K14E7 VAD, K14E7 NDR). (A) Schematic representation of the strategy used to induce severe Vitamin A Deficiency (VAD) in K14E7 transgenic model. (B) Representative H&E‐stained ectocervical sections showing CIN1–2 lesions, keratinocyte hyperproliferation (double line arrow), severe keratinization (solid black arrow), and nuclear atypia in FvB VAD mice, whereas FvB ND mice displayed normal stratified squamous epithelium, and by comparison, reduced keratinization was observed in FvB NDR mice. K14E7 ND mice exhibited basal atypia (double line arrow), mild keratinization (solid black arrow), koilocytosis (gray arrow), mixed inflammatory infiltrates (long black arrow), and CIN1‐like dysplasia. In contrast, K14E7 VAD mice developed CIN2–3 lesions characterized by marked proliferation, severe keratinization (solid black arrow), koilocytosis (gray arrow), multinucleated cells in basal to suprabasal layers (double line arrow), and moderate‐to‐severe dysplasia (dashed arrow), accompanied by dense immune infiltration (long black arrow). K14E7 NDR mice showed partial reduction of epithelial proliferation and keratinization (solid black arrow). C) Data represent cervical epithelium area. *p*‐values are shown in individual graphs: *p < 0.05, **p < 0.01, ****p < 0.0001 and ns= no statistical significance. The dashed line indicates the basal membrane. ND, Normal Diet; VAD, Vitamin A Deficient Diet; NDR, Normal Diet Reintegration; CE, Cervical Epithelium; BM, Basal Membrane; ST, Stroma.

Previous work has shown that severe Vitamin A deficiency (VAD) in the presence of the HPV‐16 E7 oncogene accelerates cervical carcinogenesis [7]; therefore, we followed a strategy to induce severe VAD in the K14E7 transgenic model (Figure 2A). Histopathological analysis (Table S1) revealed that VAD induced pronounced alterations in the cervical stratified epithelium in both FvB non‐transgenic and K14E7 transgenic mice (Figure 2B). In FvB VAD mice, squamous hyperplasia, moderate keratinization, and nuclear atypia were evident in the basal layer, whereas K14E7 VAD mice developed high‐grade intraepithelial neoplasia (CIN 2–3) with basal membrane disruption, developing stromal papillae formation, hyperkeratinization and nuclear atypia in the basal and parabasal layers. Notably, K14E7 VAD mice also displayed moderate‐to‐severe immune infiltrate, in contrast to FvB VAD and control groups. These findings highlight that E7 oncogene expression exacerbates the histopathological alterations and immune changes triggered by vitamin A deficiency. Quantitative analysis further confirmed that VAD significantly increased epithelial area in both genetic backgrounds compared with diet‐normal controls, reflecting enhanced proliferation and impaired differentiation. NDR restored epithelial morphology, reduced keratinization, and normalized epithelial area, approaching control values in FvB and partially reversing neoplastic progression in K14E7 mice (Figure 2C).

### 3.2 Cell Proliferation Levels in Ectocervix of FvB and K14E7 VAD Models Is Diminished by NDR

**Figure 3:**
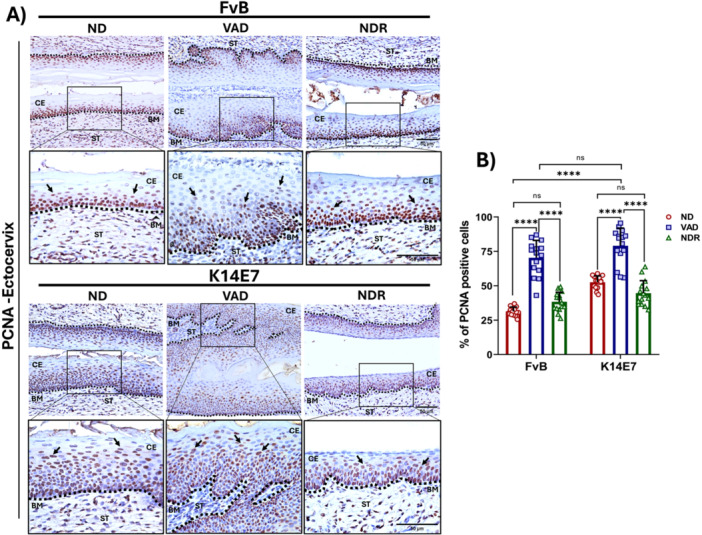
Severe vitamin A deficiency promotes enhanced cell proliferation (PCNA) within the ectocervical epithelium of 3‐month‐old FvB VAD and K14E7 VAD transgenic mice. (A) Representative PCNA photomicrographs show strong nuclear staining extending to suprabasal epithelial layers in FvB VAD and K14E7 VAD groups, whereas in controls, PCNA positive cells confined to the basal (FvB ND) and parabasal (K14E7 ND) layers. Meanwhile, FvB NDR and K14E7 NDR groups exhibited reduced staining, largely limited to basal and parabasal layers. Arrows indicate positive cells. (B) Quantification of PCNA positive cells in percentage. *p*‐values are shown in individual graphs: ****p < 0.0001 and ns = no statistical significance. The dashed line indicates the basal membrane. ND, Normal Diet; VAD, Vitamin A Deficient Diet; NDR, Normal Diet Reintegration; CE, Cervical Epithelium; BM, Basal Membrane; ST, Stroma.

To evaluate the impact of severe VAD on cervical epithelial proliferation, we assessed the nuclear expression of PCNA by IHC (Figure 3). In FvB ND mice, PCNA positivity was restricted to basal cells, whereas in K14E7 ND mice, staining extended moderately into suprabasal layers, consistent with E7‐driven proliferation. Under VAD conditions, PCNA expression markedly expanded across the epithelium. For example, FvB VAD mice showed staining in parabasal and suprabasal layers, while K14E7 VAD mice displayed nearly full‐thickness positivity, indicative of extensive proliferation (Figure 3A). Particularly, the K14E7 VAD group exhibited the highest proliferative state, demonstrating the exacerbation effect of nutritional deficiency. In contrast, both NDR groups (FvB and K14E7) showed significantly reduced proliferation, with PCNA positivity largely delimited to basal/parabasal cervical layers (Figure 3B). Collectively, these results demonstrate that severe VAD promotes an aberrant cervical epithelial proliferation, which is amplified by E7 oncoprotein, and that this proliferative phenotype is partially reversible upon reintegration of a normal diet.

### 3.3 VAD‐Induced High Cd4^+^ and CD8^+^ T‐Cell Infiltration in the Ectocervix of FvB and K14E7 VAD Models Is Not Reversed by NDR

**Figure 4:**
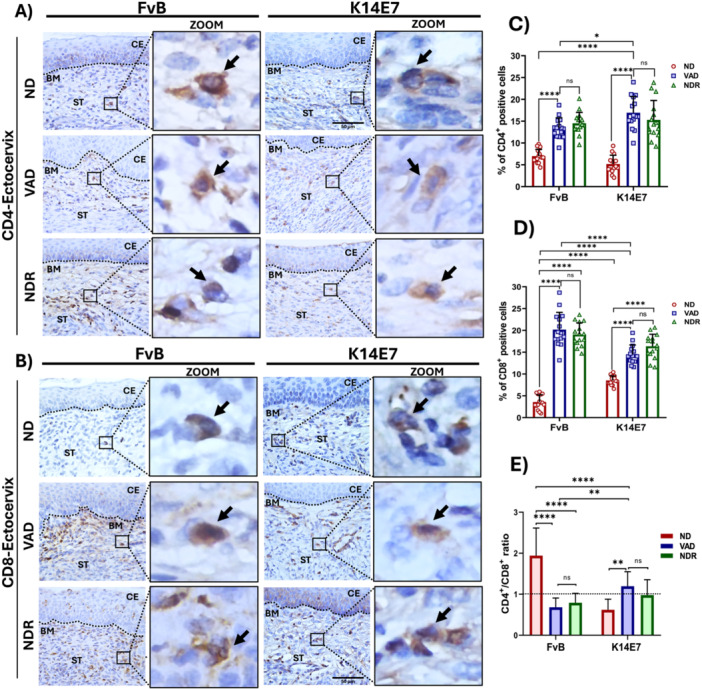
Severe Vitamin A deficiency alters CD4^+^ and CD8^+^ T cell subpopulation infiltration in the ectocervical stroma of 3‐month‐old K14E7 VAD transgenic mice. (A) Representative photomicrographs of CD4^+^ T cells and (B) CD8^+^ T cells in the cervical stroma. Both FVB VAD and K14E7 VAD mice showed increased T cell infiltration compared to their respective ND controls. FVB VAD group exhibited a robust accumulation of both CD4^+^ and CD8^+^ cells, whereas K14E7 VAD presented high CD4^+^ T cells infiltration and a relative reduction in CD8^+^ T cells. In FVB NDR and K14E7 NDR groups did not reverse CD4^+^ and CD8^+^ cell infiltration. Arrows indicate positive cells. (C, D) Quantification of CD4 and CD8 positive cells in percentage, respectively. (E) Comparison of CD4^+^/CD8^+^ ratio in FvB and K14E7 mice under different dietary conditions. *p*‐values are shown in individual graphs: *p < 0.05, **p < 0.01, ****p < 0.0001 and ns = no statistical significance. The dashed line indicates the basal membrane. ND, Normal Diet; VAD, Vitamin A Deficient Diet; NDR, Normal Diet Reintegration; CE, Cervical Epithelium; BM, Basal Membrane; ST, Stroma.

To investigate the effects of severe VAD on adaptive immunity, we quantified the infiltration of CD4^+^ (Figure 4A) and CD8^+^ T cells (Figure 4B). In FvB control mice, the levels of CD4^+^ and CD8^+^ T cells were low. However, the K14E7 ND mice moderately increased T‐cell infiltration, indicating that E7 oncoprotein by itself modifies the local immune microenvironment. Meanwhile, VAD groups led to a marked expansion of both T‐cell populations in both FvB and K14E7 mice. Notably, the K14E7 VAD group exhibited a shift toward higher CD4^+^ and lower CD8^+^ cell infiltration, suggesting that E7, under these nutritional deficiency conditions, exacerbates immune dysregulation and promotes an immunosuppressive mechanism. Remarkably, the NDR group demonstrated a mild migration of CD8^+^ T cells into the epithelium. Nonetheless, overall T‐cell infiltration remained elevated in the stromal sub‐epithelium and cervical stroma, with no significant differences observed between both VAD models (FvB and K14E7), probably due to the persistent proinflammatory state induced by VAD (Figure 4C,D). The CD4^+^/CD8^+^ ratio was significantly reduced in FvB VAD compared with ND controls was not restored following dietary normalization. In K14E7 mice, the ratio was altered under ND conditions relative to FvB controls and increased further under VAD. NDR partially shifted the ratio toward ND values, although complete normalization was not observed (Figure 4E).

### 3.4 NDR Restored Vad‐Induced Cytotoxic Dysfunction by Increasing Gzmb and Reducing PI‐9 in the Ectocervix of FvB and K14E7 VAD Models

**Figure 5:**
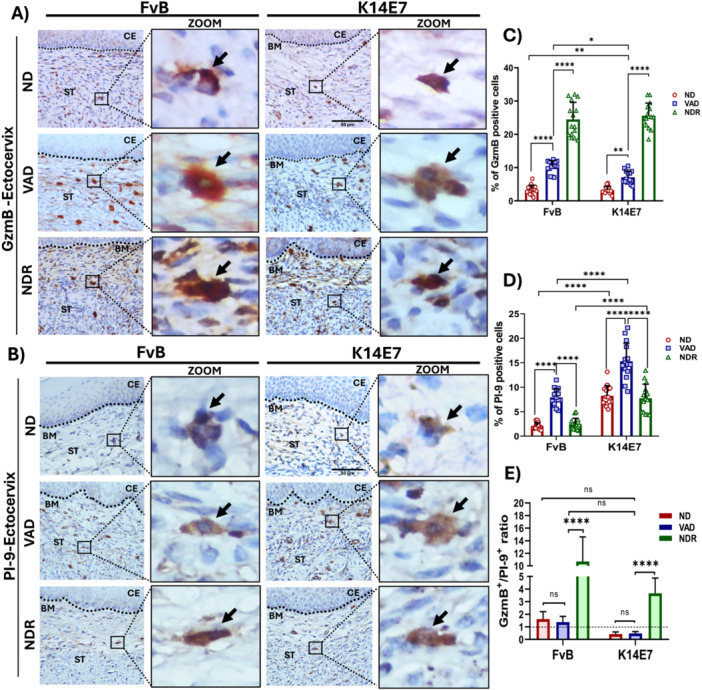
Severe Vitamin A deficiency induces cytotoxic imbalance via upregulation of intracellular PI‐9 despite GzmB activity in the ectocervical stroma of 3‐month‐old FvB VAD and K14E7 VAD transgenic mice. (A) Representative photomicrographs of GzmB and B) PI‐9 in the cervical stroma. FVB VAD exhibited an increase in GzmB+ cells compared with FVB ND. In contrast, K14E7 VAD showed reduced GzmB+ cells. PI‐9, was upregulated in VAD groups, with the highest expression in K14E7 VAD. Conversely, NDR groups exhibited increased numbers of GzmB+ cells and reduced PI‐9 expression. Arrows indicate positive cells. (C, D) Quantification of CD4 and CD8 positive cells in percentage, respectively. (E) Comparison of GzmB + /PI‐9+ ratio in FvB and K14E7 mice under different dietary conditions. *p*‐values are shown in individual graphs: *p < 0.05, **p < 0.01, ****p < 0.0001 and ns = no statistical significance. The dashed line indicates the basal membrane. ND, Normal Diet; VAD, Vitamin A Deficient Diet; NDR, Normal Diet Reintegration; CE, Cervical Epithelium; BM, Basal Membrane; ST, Stroma.

Since NDR did not alter CD4^+^ or CD8^+^ T‐cell infiltration, we assessed whether cytotoxic function was affected by evaluating GzmB (Figure 5A) and its endogenous inhibitor PI‐9 (Figure 5B) expression. Under ND conditions, FvB mice exhibited low GzmB and minimal PI‐9 expression, whereas K14E7 mice showed slightly higher GzmB and PI‐9 expression, reflecting basal cytotoxic activity and modest immune modulation by E7. On the other hand, VAD groups induced a strong upregulation of GzmB and PI‐9 in both models (FvB VAD and K14E7 VAD), indicating a cytotoxic immunoregulatory environment. Strikingly, the K14E7 VAD group showed the highest levels of PI‐9 than FvB VAD, suggesting that HPV‐16 E7 oncoprotein constrains cytotoxic responses while reinforcing immune evasion. In contrast, NDR partially restored cytotoxic balance in both models (FvB NDR and K14E7 NDR), with significantly increased GzmB and reduced PI‐9 expression, although not to basal ND levels (Figure 5C,D). Particularly, in K14E7 mice, NDR produced a moderate reduction in PI‐9, highlighting the effect of E7‐driven immune modulation. The GzmB^+^/PI‐9^+^ ratio was reduced in K14E7 ND mice compared with FvB controls and decreased further under VAD. Dietary normalization increased this ratio in K14E7 mice, partially restoring it toward control levels. In FvB mice, the ratio remained relatively stable under VAD and was markedly elevated following dietary normalization (Figure 5E).

### 3.5 VAD Induced High Foxp3^+^ Cells and Macrophage F4/80^+^ Infiltration in the Ectocervix of FvB and K14E7 VAD Models

**Figure 6:**
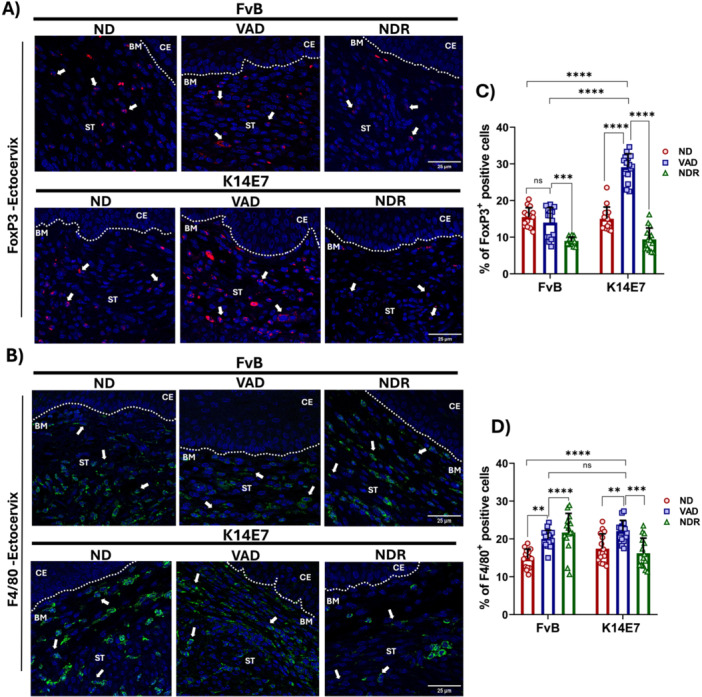
Severe Vitamin A deficiency increases FoxP3^+^ cells and macrophage F4/80^+^ infiltration in the ectocervical stroma of 3‐ month‐old FvB VAD and K14E7 VAD transgenic mice. (A) Representative photomicrographs of FoxP3^+^ cells and (B) F4/80^+^ macrophages in the cervical stroma. FoxP3^+^ regulatory T cells were significantly increased under VAD conditions in K14E7 mice compared with FvB VAD and their respective ND controls. Following NDR, FoxP3 expression was reduced in both models. In parallel, both FvB VAD and K14E7 VAD mice exhibited a significant increase in macrophage infiltration compared with ND controls, with the highest levels again observed in K14E7 VAD mice. In FvB mice, NDR induced a mild increase in macrophage numbers, whereas in K14E7 mice it resulted in a reduction compared with VAD conditions. (C, D) Quantification of FoxP3 and F4/80 positive cells in percentage, respectively. *p*‐values are shown in individual graphs: **p < 0.01, ***p < 0.001, ****p < 0.0001 and ns = no statistical significance. The dashed line indicates the basal membrane. ND, Normal Diet; VAD, Vitamin A Deficient Diet; NDR, Normal Diet Reintegration; CE, Cervical Epithelium; BM, Basal Membrane; ST, Stroma.

Immunofluorescence analysis revealed a low basal presence of FoxP3^+^ regulatory T cells in the stromal compartment of FvB mice under ND. In contrast, VAD induced a clear increase in FoxP3^+^ cells, more pronounced in K14E7 mice. Notably, the highest accumulation of FoxP3^+^ cells was observed in the K14E7 VAD group, indicating a synergistic effect between HPV16 E7 expression and Vitamin A deficiency in promoting Treg expansion. Following NDR, FoxP3 expression was significantly reduced in both models (FvB and K14E7), although not fully restored to basal levels (Figure 6A). Quantitative analysis confirmed these observations, demonstrating a significant increase in FoxP3^+^ cells in K14E7 VAD (Figure 6C). These findings indicate that VAD promotes a Treg‐enriched immunosuppressive microenvironment, particularly in the presence of HPV16 E7.

Macrophages are key regulators of the tumor microenvironment. Thus, we assessed F4/80^+^ macrophage infiltration in ectocervical stroma (Figure 6B). In the FvB ND group, moderate numbers of F4/80^+^ macrophages were observed, reflecting basal immune cell presence. Meanwhile, VAD led to a modest increase in macrophage infiltration, whereas NDR reduced this infiltration to intermediate levels, suggesting persistent residual effects of Vitamin A deficiency. In K14E7 mice, macrophage infiltration was elevated across all dietary conditions, indicating that E7 expression is associated with sustained macrophage recruitment, which is further increased under VAD conditions (Figure 6D).

## 4 Discussion

Persistent HR‐HPV infection is linked to the development of a chronic inflammatory microenvironment, which promotes epithelial changes, and increased immune cell infiltration features observed in human cervical intraepithelial lesions [17, 18]. Previously, various risk factors that contribute to the progression and development of CC have been identified such as Vitamin A deficiency [19, 20]. Accordingly, the transgenic model K14E7 [16] is an ideal preclinical tool to mimic chronic HPV‐induced infection. Our histopathological findings confirm these alterations in the ectocervical epithelium of the K14E7 DN group confirming early dysplastic changes associated with E7 expression [21]. However, we observed that VAD significantly increased these changes in cervical tissue, suggesting that nutritional status acts as a critical modulator of the inflammatory microenvironment and neoplastic progression. Therefore, in the presence of both factors (expression of HPV‐16 E7 oncoprotein and VAD), progression to high‐grade intraepithelial lesions (CIN II‐III) is promoted, with increase keratinization and immune cell infiltration in the cervical epithelium and stroma of the K14E7 VAD group (see Figure 2B). These results align with those previously reported, demonstrating a synergistic effect between nutritional deficiency and the oncogenic activity of E7 [11]. Interestingly, in the groups where the normal diet was resumed during the last month of age (FvB NDR and K14E7 NDR), after a Vitamin A deficiency during 2 months, they showed a reduced dysplasia and partially restored epithelial; suggesting that retinol can partially reverse E7 expression and VAD‐induced alterations.

Partial recovery of NDR suggests persistence of vitamin A‐dependent regulatory mechanisms for epithelial health and immune regulation remain active even after deficiency. Incomplete recovery likely reflects limited reintegration duration. Longer reintegration of Vitamina A (retinoid) exposure [22, 23] may enhance tissue organization and reduce disease alterations. Additionally, persistent HPV16 E7 expression may impede full recovery due to ongoing oncoviral changes. Therefore, further research on prolonged vitamin A reintroduction is crucial for assessing its impact on HPV‐associated cervical disease severity. From a translational perspective, epidemiological studies support an association between vitamin A status and HPV‐related cervical disease. Although data in HPV‐positive individuals from low‐income settings remain limited, available evidence indicates that low dietary intake of retinol or serum retinol levels are associated with increased risk of HPV infection and cervical neoplasia, whereas vitamin A supplementation or higher intake appears protective against cervical intraepithelial neoplasia and invasive cervical cancer [24, 25, 26]. Together, these studies support a protective association between adequate Vitamin A status and HPV‐related cervical disease. Although these observational findings do not establish causality, they reinforce the mechanistic framework proposed in the present study, suggesting that retinoic acid–dependent regulation of epithelial differentiation and immune responses may influence HPV persistence and cervical carcinogenesis. Thus, the possibility of using Vitamin A or a synthetic retinoid supplementation as an adjuvant to control low grade cervical lesions in low‐income populations may not be so far away.

In addition to histopathological, E7‐induced cell cycle dysregulation was evidenced by increased cell proliferation in the K14E7 DN group, likely mediated by pRb inactivation [27] and p21^Cip1 interference, promoting G1‐S progression [28]. This proliferative effect was amplified in the K14E7 VAD group, indicating that Vitamin A deficiency further disrupts control over cell proliferation. In contrast, the FvB NDR and K14E7 NDR groups showed a significant decrease in proliferation, with PCNA expression restricted to the basal epithelial layer. This supports the modulatory role of Vitamin A in controlling cell proliferation and epithelial homeostasis [29], partly mediated by RARβ tumor suppressor [30].

Our analysis of immune infiltration revealed increased recruitment of CD4^+^ and CD8^+^ T lymphocytes in the ectocervical microenvironment of K14E7 VAD mice, with a predominance of CD4^+^ T lymphocytes. This pattern is consistent with clinical studies reporting a progressive increase in infiltrating T cells as cervical lesions advance, particularly with a higher presence of CD4+ compared to CD8+ in severe dysplasia stages [31, 32]. Notably, CD4^+^/CD8^+^ ratio was increased, confirming CD4^+^ T cells predominance under these conditions. One possible explanation is the ability of HVP16 E7 to interfere with antigen presentation, as it reduces the expression of MHC class I‐associated genes in transformed epithelial cells [33], thereby limiting CD8^+^ T cell activation and promoting functional exhaustion, which may amplify VAD‐induced immune dysregulation and unbalance T helper CD4^+^ (Th cells) subpopulations, including Tregs [34]. This infiltration pattern was not fully reversed by NDR, indicating that nutritional reintegration was not sufficient to normalize the immunological microenvironment. In agreement with the altered balance of CD4^+^ and CD8^+^ T cells, we observed increased expression of FoxP3 in K14E7 VAD mice, indicating the presence of regulatory T cells (Tregs) in the cervical microenvironment. Tregs are known to suppress antitumor immune responses by limiting the activity of effector T cells [35]. In the context of HPV‐associated carcinogenesis, studies have reported an increased infiltration of FoxP3^+^ Tregs in cervical lesions and tumors, where they contribute to creating an immunoregulatory niche that promotes viral persistence and tumor progression [36, 37]. Thus, the elevated FoxP3 expression observed in K14E7 VAD mice may contribute to an immunoregulatory microenvironment associated with ineffective immune surveillance, HPV persistence and tumor progression. Interestingly, in NDR groups, FoxP3 expression, was partially reduced.

Although the combined presence of E7 expression and VAD promotes recruitment of T lymphocytes to the cervical tissue, the cytotoxic functionality of CD8^+^ T cells is compromised. CD8^+^ T lymphocytes can exert their cytotoxic action against tumor cells by employing different apoptosis induction strategies. A direct method is mediated by the release of perforins and granzymes, which leads to the activation of various caspases [38]. While the synergy of both factors studied (E7 and VAD) represents a scenario that favors the recruitment of CD4^+^ and CD8^+^ T lymphocytes at the subepithelial and stromal levels of the cervix, the cytotoxic functionality of CD8^+^ T cells compromised, is reflected by the low expression of GzmB and overexpression of its endogenous inhibitor, PI‐9. Consistent with a CD8^+^ T cell exhaustion phenotype reported in cervical cancer [39] or induced by chronic exposure to E7 [40].

Elevated expression of PI‐9 has been reported in CC, correlating with disease stage [41] serving as a mechanism of immune evasion employed by tumor cells. Consistent with this, previous studies using the K14E7 model, showed that E7, along with estrogens, negatively regulates GzmB and increases PI‐9 in keratinocytes [42]. Although the NDR did not increase the lymphocytic infiltrate, it partially restored effector capacity by increasing GzmB and a reduction in PI‐9, suggesting that RA‐mediated signaling restores the cytotoxic differentiation of CD8^+^ T lymphocytes [43]. This effect could be explained by the participation of nuclear receptors such as RARγ, which has been associated with the induction of an effector response, such as the formation of memory CD8^+^ T lymphocytes. However, the mechanism has not yet been clarified [44]. These findings are consistent with some reports highlighting the need for RA to acquire an effector phenotype in CD8^+^ T cells, characterized by high expression of GzmB [45]. Thus, our results suggest that reintegration of Vitamin A reprograms CD8^+^ T cell cytotoxic function and may limit an immunosuppressive tumor microenvironment, thereby restraining cervical carcinogenesis.

In addition to lymphocyte alterations, we observed increased infiltration of F4/80+ macrophage in both FvB VAD, and K14E7 VAD mice compared with control groups (FvB DN and K14E7 DN). In FvB VAD mice with lesions equivalent to CIN I‐II, an increase in macrophage infiltrate was observed in the ectocervix stroma. This observation is consistent with clinical studies reporting enhanced tumor‐associated macrophages (TAMs) infiltration within tumor microenvironment of CIN I‐III and invasive CC [46], indeed, it could be enhanced by VAD conditions, suggesting its immunomodulatory activity. Meanwhile, K14E7 VAD mice, showed the highest macrophage infiltrate, related to oncoprotein E7 in enhancing inflammation. The increased F4/80+ macrophage infiltrate in FvB VAD and K14E7 VAD could be associated with IFN‐γ – driven chemokine signaling described in VAD [47] The NDR group showed increased F4/80+ macrophages infiltration in FvB, possibly related to tissue repair and RA‐mediated macrophage polarization [48]. In K14E7, Vitamin A reintegration could modulate NF‐κB‐dependent pro‐inflammatory pathways [49], partially attenuating the F4/80+ macrophage infiltration.

### 4.1 Study Limitations

Some limitations of the present study should be acknowledged. Although F4/80 staining demonstrated increased macrophage infiltration, this marker does not distinguish between pro‐inflammatory and immunosuppressive macrophage subsets. Therefore, the increased macrophage abundance observed in both VAD groups likely reflects enhanced immune cell recruitment rather than definitive functional polarization. In HPV‐driven carcinogenesis, immune cell infiltration may coexist with impaired cytotoxic responses, contributing to an immunologically ineffective microenvironment. Additionally, circulating serum retinol levels were not directly quantified; therefore, the severity of Vitamin A deficiency was inferred from the dietary intervention protocol previously validated in comparable K14E7 experimental models. Likewise, local cytokine profiling and functional characterization of macrophage polarization (M1‐like vs M2‐like) were not performed, limiting a more comprehensive understanding of the inflammatory and immunoregulatory cervical microenvironment under different dietary conditions.

Future studies incorporating biochemical quantification of serum retinol and retinoic acid metabolites, together with cytokine profiling and macrophage subset characterization, would further strengthen the translational relevance and mechanistic interpretation of these findings.

## 5 Conclusion

In conclusion, our results demonstrate that the interaction between the HPV‐16 E7 oncoprotein and severe vitamin A deficiency disrupts the cervical microenvironment, promoting epithelial dysplasia and an immune profile characterized by increased CD4^+^ and CD8^+^ T‐cell and F4/80^+^ macrophage infiltration, yet impaired cytotoxic function. This imbalance is evidenced by alterations in the CD4^+^/CD8^+^ ratio, increased FoxP3 expression, and a reduced GzmB^+^/PI‐9^+^ ratio. Together, these findings indicate that vitamin A is a critical regulator of local adaptive immunity and E7‐driven neoplastic progression. Importantly, vitamin A reintegration partially restores epithelial architecture and CD8^+^ T‐cell effector function. Thus, the K14E7 VAD model illustrates how viral and nutritional factors cooperate to promote early neoplastic progression, supporting vitamin A restoration as a potential strategy to modulate immune responses in HPV‐associated cervical cancer.

## Author Contributions

All authors contributed to the study conception and design. Patricio Gariglio and Solangy Lizcano‐Meneses advised the development of this work. Armando Chávez‐Ríos, Celina López‐Ruiz, Ian A. García‐Aguirre, Rodolfo Ocadiz‐Delgado, Francisco Garcia‐Sierra, Solangy Lizcano‐Meneses and Patricio Gariglio contributed to data collection and analysis of the results. The maintenance and care of murine strains (FvB non‐transgenic and K14E7 transgenic) and experimental procedures were performed by Armando Chávez‐Ríos, Celina López‐Ruiz and Solangy Lizcano‐Meneses. The histopathological analysis of ectocervical tissue of murine strains was performed by Rogelio Hernández‐Pando. The first draft of the manuscript was written by Armando Chávez‐Ríos, Solangy Lizcano‐Meneses and Patricio Gariglio. All authors read and approved the final manuscript.

## Ethics Statement

All animal experiments were performed in accordance with the Association for Assessment and Accreditation of Laboratory Animal Care International (AAALAC) guidelines and were approved by the Institutional Committee for Laboratory Animal Research and Care (UPEAL‐CINVESTAV‐IPN, Mexico; NOM‐062‐ZOO‐1999).

## Conflicts of Interest

The authors declare no conflicts of interest.

## Supporting information


**Figure S1:** Schematic representation of the dietary regimens and proposed cervical immune microenvironment in the K14E7 preclinical model.
**Table S1:** Histopathological analysis of ectocervical tissue in 3‐month‐old non‐transgenic FvB and K14E7 transgenic mice under normal diet (ND), severe Vitamin A deficiency (VAD) or normal diet reintegration (NDR) conditions.
**Table S2:** Antibodies used in IHCs.
**Table S3:** Antibody used in IF.

## Data Availability

The data that support the findings of this study are available from the corresponding author upon reasonable request.
