# Supplementary material for: Synergistic Interaction Between HPV‐16 E7 Oncoprotein and Severe Vitamin A Deficiency in Regulating Adaptive Immunity in a Preclinical Cervical Cancer Model
Source: J Med Virol. 2026 Jun 10;98(6):e70992. doi: 10.1002/jmv.70992 (PMC13250677; doi:10.1002/jmv.70992)
Supplement: Supplementary file 1 — Figure S1: Schematic representation of the dietary regimens and proposed cervical immune microenvironment in the K14E7 preclinical model. Table S1: Histopathological analysis of ectocervical tissue in 3‐month‐old non‐transgenic FvB and K14E7 transgenic mice under normal diet (ND), severe Vitamin A deficiency (VAD) or normal diet reintegration (NDR) conditions. Table S2: Antibodies used in IHCs. Table S3: Antibody used in IF. [file JMV-98-e70992-s001.pdf]

SUPPLEMENTARY SECTION

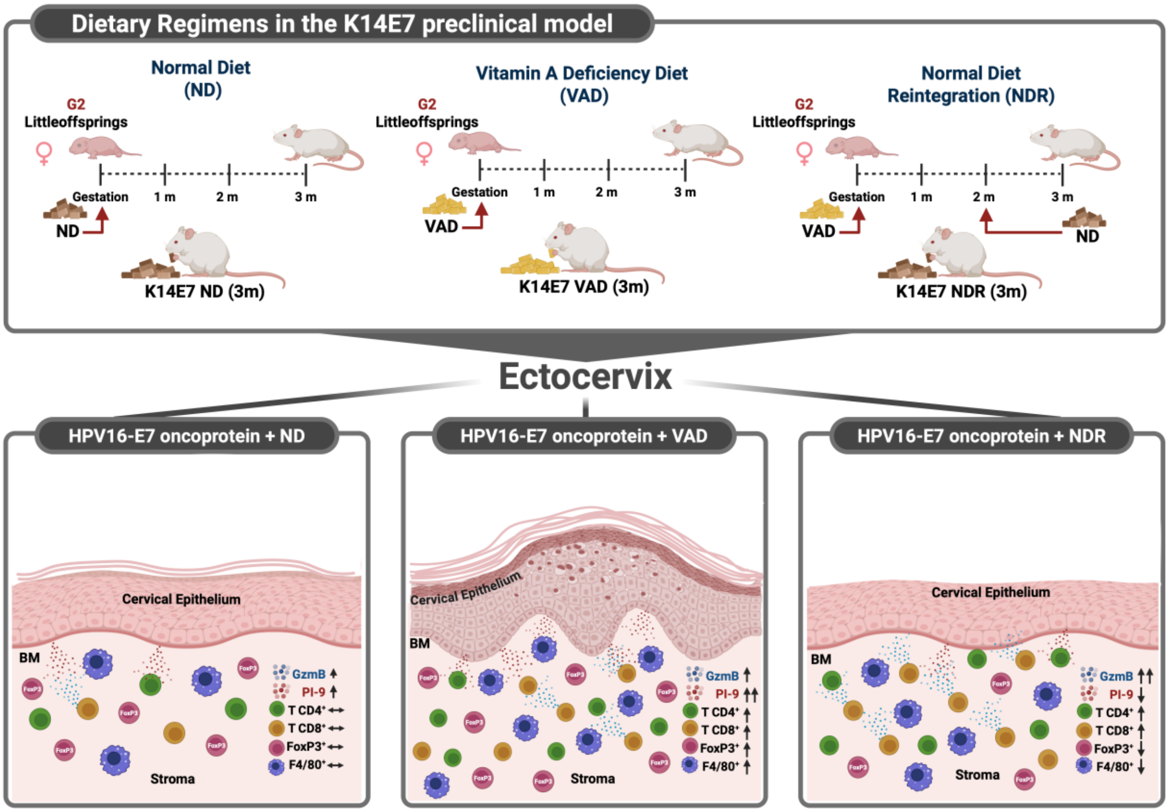

Supplementary Figure 1. Schematic representation of the dietary regimens and proposed cervical immune microenvironment in the K14E7 preclinical model. Upper panel: experimental design of normal diet (ND), vitamin A-deficient diet (VAD), and normal diet reintegration (NDR) conditions in K14E7 mice from gestation to 3 months of age. Lower panel: proposed effects of HPV16 E7 expression combined with VAD on cervical epithelial architecture and immune cell infiltration. VAD promoted epithelial thickening, increased inflammatory and immunoregulatory cell infiltration, and reduced GzmB expression, whereas NDR partially restored cytotoxic immune balance. Immune populations evaluated include $CD4^+$ T cells, $CD8^+$ T cells, $FoxP3^+$ cells, $F4/80^+$ macrophages, $PI\text{-}9^+$ cells, and $GzmB^+$ cells. BM: basal membrane.

Supplementary Table 1. Histopathological analysis of ectocervical tissue in 3-month-old non-transgenic FvB and K14E7 transgenic mice under normal diet (ND), severe Vitamin A deficiency (VAD) or normal diet reintegration (NDR) conditions.

Genotype with dietary regimens	Histopathological analysis	Consistency (%)
**FvB (ND) (n = 3)**	Normal epithelium.	100
**FvB (VAD) (n = 3)**	Moderate dysplasia (CIN 2). Dysplastic nuclei in the basal stratum.Mild presence of inflammatory infiltrate.	100
**FvB (NDR) (n = 3)**	Normal epithelium. Occasional dysplastic cells. Scanty inflammatory infiltrate.	100
**K14E7 (ND) (n = 3)**	Mild dysplasia. Dysplastic cells in the basal stratum. Mild presence of inflammatory infiltrate	100
**K14E7 (VAD) (n = 3)**	Moderate and severe dysplasia (CIN 2-3). Presence of dysplastic cells in various stratums of the epithelium. Increased inflammatory infiltrate	100
**K14E7 (NDR) (n = 3)**	Mild to moderate dysplasia (CIN 1-2). Dysplastic cells in the different stratums of the epithelium. Mild presence of inflammatory infiltrate	100

Supplementary Table 2. Antibodies used in IHCs

N°	Antibody	Type	Isotype	Dilution	Species	Trademark
**1**	PCNA	Monoclonal	IgG2aκ	1:50	Mouse	Santa Cruz Biotechnology sc-25280
**2**	GzmB	Polyclonal	IgG	1:50	Rabbit	Abcam / ab4059
**3**	PI-9	Monoclonal	IgG1	1:50	Mouse	Santa Cruz Biotechnology sc-57531
**4**	CD4	Monoclonal	IgG	1:50	Rabbit	Cell Signaling Technology / D7D2Z
**5**	CD8	Polyclonal	IgG	1:50	Mouse	Santa Cruz Biotechnology sc-1177

Supplementary Table 3. Antibody used in IF.

N°	Antibody	Type	Isotype	Dilution	Species	Trademark
**1**	F4/80	Monoclonal	IgG2b	1:100	Rat	Abcam / ab6640
**2**	FoxP3	Monoclonal	IgG	1:100	Rabbit-Conjugated APC	R&D SYSTEMS/ IC8214A
